# Dual RNA-Seq Analysis Pinpoints a Balanced Regulation between Symbiosis and Immunity in *Medicago truncatula*-*Sinorhizobium meliloti* Symbiotic Nodules

**DOI:** 10.3390/ijms242216178

**Published:** 2023-11-10

**Authors:** Dandan Zhang, Qiujin Wu, Yanwen Zhao, Ziang Yan, Aifang Xiao, Haixiang Yu, Yangrong Cao

**Affiliations:** National Key Laboratory of Agricultural Microbiology, College of Life Science and Technology, Huazhong Agricultural University, Wuhan 430070, China; zdd0103@webmail.hzau.edu.cn (D.Z.);

**Keywords:** legume–rhizobial symbiosis, *Medicago truncatula*, *Sinorhizobium meliloti*, *NAD1*, plant defense response, dual RNA-seq

## Abstract

Legume–rhizobial symbiosis initiates the formation of root nodules, within which rhizobia reside and differentiate into bacteroids to convert nitrogen into ammonium, facilitating plant growth. This process raises a fundamental question: how is plant immunity modulated within nodules when exposed to a substantial number of foreign bacteria? In *Medicago truncatula*, a mutation in the *NAD1* (*Nodules with Activated Defense 1*) gene exclusively results in the formation of necrotic nodules combined with activated immunity, underscoring the critical role of *NAD1* in suppressing immunity within nodules. In this study, we employed a dual RNA-seq transcriptomic technology to comprehensively analyze gene expression from both hosts and symbionts in the *nad1-1* mutant nodules at different developmental stages (6 dpi and 10 dpi). We identified 89 differentially expressed genes (DEGs) related to symbiotic nitrogen fixation and 89 DEGs from *M. truncatula* associated with immunity in the *nad1-1* nodules. Concurrently, we identified 27 rhizobial DEGs in the *fix* and *nif* genes of *Sinorhizobium meliloti*. Furthermore, we identified 56 DEGs from *S. meliloti* that are related to stress responses to ROS and NO. Our analyses of nitrogen fixation-defective plant *nad1-1* mutants with overactivated defenses suggest that the host employs plant immunity to regulate the substantial bacterial colonization in nodules. These findings shed light on the role of *NAD1* in inhibiting the plant’s immune response to maintain numerous rhizobial endosymbiosis in nodules.

## 1. Introduction

Nitrogen is one of the necessary nutrient elements required for plant growth and development [1,2]. However, nitrogen in nature can only be absorbed by plants after being reduced into nitrate or ammonia nitrogen [2,3]. Nodule symbiosis plays an important role in reducing nitrogen into ammonium for host plants [4]. In the legume–rhizobial symbiosis, the compatible interaction between rhizobium and legume roots results in the formation of root nodules, where rhizobium resides and develops into bacteroids inside of symbiotic cells, to convert nitrogen into organic nitrogen compounds for plant growth and development [5,6,7]. The large colonization of rhizobia inside symbiotic cells raises an opening question as to how plant immunity is attenuated in symbiotic cells with the residence of foreign bacteria.

*Medicago truncatula* is a model legume that has long been used for the study of rhizobial symbiosis. The nodules produced on *M. truncatula* are morphologically classified as indeterminate nodules, with four different zones identified. Zone I is the persistent meristem at the distal part of the root nodule; Zone II is the infection zone that rhizobia releases from the infection thread into host cells; Zone II–III is the transition zone; Zone III is the nitrogen-fixing area; and Zone IV is the senescent zone, where bacteria and host cells are to be degraded [8,9,10]. In addition, the bacteroids in *M. truncatula* nodules undergo terminal differentiation, including cell expansion, genome endoreduplication, membrane modification, and loss of reproductive ability, which is largely due to the presence of the large amount of Nodule-specific Cysteine-Rich (NCR) peptides from host plants [11,12,13,14]. However, the function of NCR peptides in regulating nodulation is largely unclear.

There is an exchange of chemical signals between plants and rhizobia throughout the different stages of nodule development, including nodule formation, bacterial recognition, and development of bacterial infection, which encompasses Nod factor signaling (NF signaling) as well as other plant signaling systems involving calcium, NADPH oxidase, and NO synthase systems [15,16,17,18,19]. These systems, inducing alterations in gene expression within both partners, play a role in shaping partner selection and dampening plant defenses [20,21]. These signals facilitate the entry of bacteria into plant epidermal and cortical cells, stimulating root cell division and the development of nodule meristem, leading to the formation of numerous specialized cellular organelles referred to as “symbiosomes”, each housing one or more nitrogen-fixing bacteroids [17,20].

The role of plant innate immunity in regulating plant associations with different microbes, including beneficial microbes, has drawn great attention from scientists worldwide. Microbe-associated molecular patterns (MAMPs) derived from pathogens can be recognized by pattern recognition receptors (PRRs) on plant cell membranes to trigger a variety of early defense reactions, including the production of a large number of reactive oxygen species (ROS) [17,22]. At present, the study of PRRs mainly involves bacterial flagellin receptor FLAGELLIN SENSING 2 (FLS2) and chitin elicitor receptor kinase (CERK1) [23,24,25]. In order to overcome the PAMP-triggered immune response (PTI), pathogens secrete effector proteins into plant cells through a type III secretion system to inhibit plant immunity. Therefore, plants have evolved a variety of disease resistance (R) proteins to recognize effectors and produce effector-triggered immunity (ETI) [26,27]. Most of these R proteins belong to nucleoside binding site–leucine rich repeat (NBS–LRR) [28,29]. The activation of a large number of defense genes in ETI is often accompanied by hypersensitivity or hypersensitivity-like cell death and finally induces resistance to pathogens [30,31]. In fact, similar to pathogens invading host plants, rhizobia can induce host plant innate immunity in plants when they come in contact with the roots of legume plants. However, this host defense response is transient, and then it is gradually inhibited during legume–rhizobial symbiotic establishment [32]. Symbiosis-induced immunosuppression is regulated by rhizobia or host plants in a variety of manners. Many pieces of evidence have indicated that rhizobia have evolved multiple strategies, including ROS scavenging enzymes, nodulation factors (NFs), lipopolysaccharides (LPS), extracellular polysaccharides (EPS), and type III/IV secretion systems to escape or inhibit the immunity produced by the early symbiotic host [33,34,35].

Despite the massive invasion of the rhizobia released from the infection thread, the symbiotic cells do not display excessive activation of defense response. For example, in terms of host plants, multiple *M. truncatula* genes such as *Defective in Nitrogen Fixation 2* (*DNF2*) [36], *Symbiotic Cysteine-Rich Receptor-Like Kinase* (*SymCRK*), [37], *Regulator of Symbiosome Differentiation* (*RSD*) [38], and *Nodules with Activated Defense 1* (*NAD1*) [39] result in the formation of defective nodules with over-activated defense responses and/or premature phenotypes. Non-fixing nitrogen nodules in these mutants show necrotic cells with typical defense features such as the accumulation of phenolic compounds and over-activation of defense-related genes (*PR10* and *NADR1*) [36,37,39,40]. However, intriguingly, the function of the above genes required for nodule immunity is currently reported only in *M. truncatula*. Thus, the *M. truncatula* nodule serves as an ideal model for studying how plant immunity regulates rhizobial residence in symbiotic cells.

Our previous work identified that *M. truncatula NAD1* is an indispensable component for maintaining rhizobial colonization by preventing immune responses in nodules [39,40,41]. To gain further insights into the mechanism by which plant immunity regulates symbiotic interaction with symbionts in *M. truncatula* nodules, a dual RNA-seq was performed to present a landscape of how hosts and symbionts simultaneously cooperate with each other at transcriptional levels in the *M. truncatula nad1* nodules.

## 2. Results and Discussion

### 2.1. M. truncatula NAD1 Is Indispensable for Nodule Development

**Figure 1:**
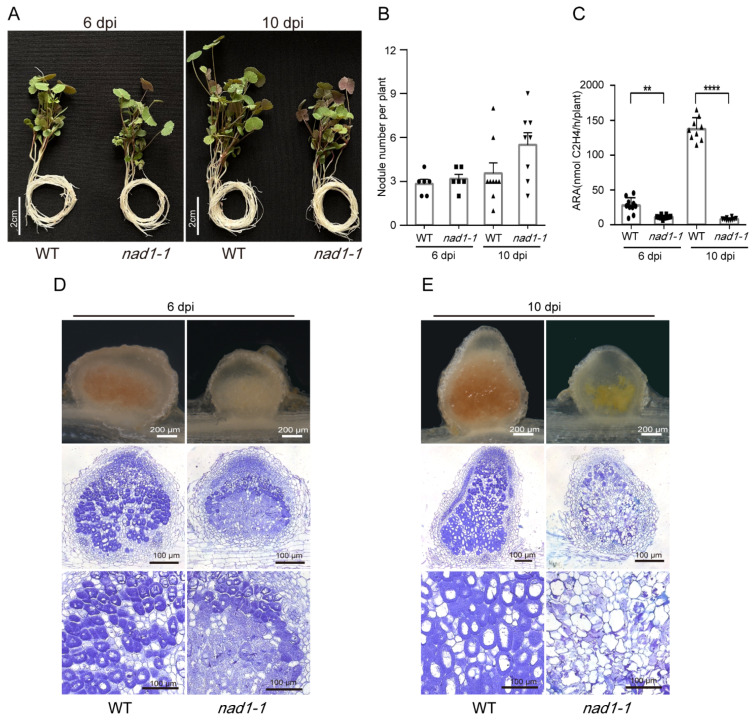
Phenotypes of the wild-type (WT) *Medicago truncatula* R108 and *nad1-1* mutant. (**A**) Growth of WT and *nad1-1* at 6 days post-inoculation (dpi) and 10 dpi inoculated with *Sinorhizobium meliloti* 2011. (**B**) Nodule number per plant was measured at 6 dpi and 10 dpi. (**C**) Acetylene reduction assay (ARA) reflecting nitrogenase activity was performed on nodulated plants. **, *p* < 0.05; ****, *p* < 0.01. (**D**,**E**) Sections of WT and *nad1-1* mutant nodules at 6 dpi and 10 dpi.

*M. truncatula nad1* mutant plants produce necrotic nodules with activated defense responses [39,40]. To explain the detailed mechanisms of how hosts interact with symbionts in *M. truncatula nad1* nodules, we set up experiments using the young nodules from *M. truncatula nad1* plants. At 6 days post-inoculation (dpi) and 10 dpi with rhizobium, no significant difference in plant growth was observed between the *nad1-1* mutants and the wild-type *M. truncatula* plants (Figure 1A). The number of root nodules in the *nad1-1* mutants showed no difference from that in the wild type at 6 dpi, while it was higher than that in the wild type at 10 dpi (Figure 1B). Furthermore, the nitrogenase activity in *nad1-1* mutants was significantly lower than that in the wild type at both 6 and 10 dpi (Figure 1C). Compared to the absence of brown pigments observed in the *nad1-1* nodules at 6 dpi, significant deposition of severe brown pigments was detected in the *nad1-1* nodules at 10 dpi, in contrast to the pink nodules of the wild type (Figure 1D,E). This observation indicates that the defense responses in *nad1-1* nodules become exaggerated along with the development of the nodules. At 6 dpi, we observed abnormal infected cells with a reduced population of bacteria in Zone III in the *nad1-1* nodules compared to the wild-type nodules (Figure 1D). However, at 10 dpi, almost no intact symbiotic cells were observed in Zone III of the *nad1-1* nodules. Instead, bacteroids were released from the cells and accumulated in non-symbiotic intercellular spaces within the *nad1-1* mutant nodules (Figure 1E). These results indicated that *NAD1* is essential for the development of plant root nodules, nitrogen-fixing ability, and the survival and colonization of rhizobium.

### 2.2. Identification of DEGs from Hosts in the M. truncatula nad1 Nodules

**Figure 2:**
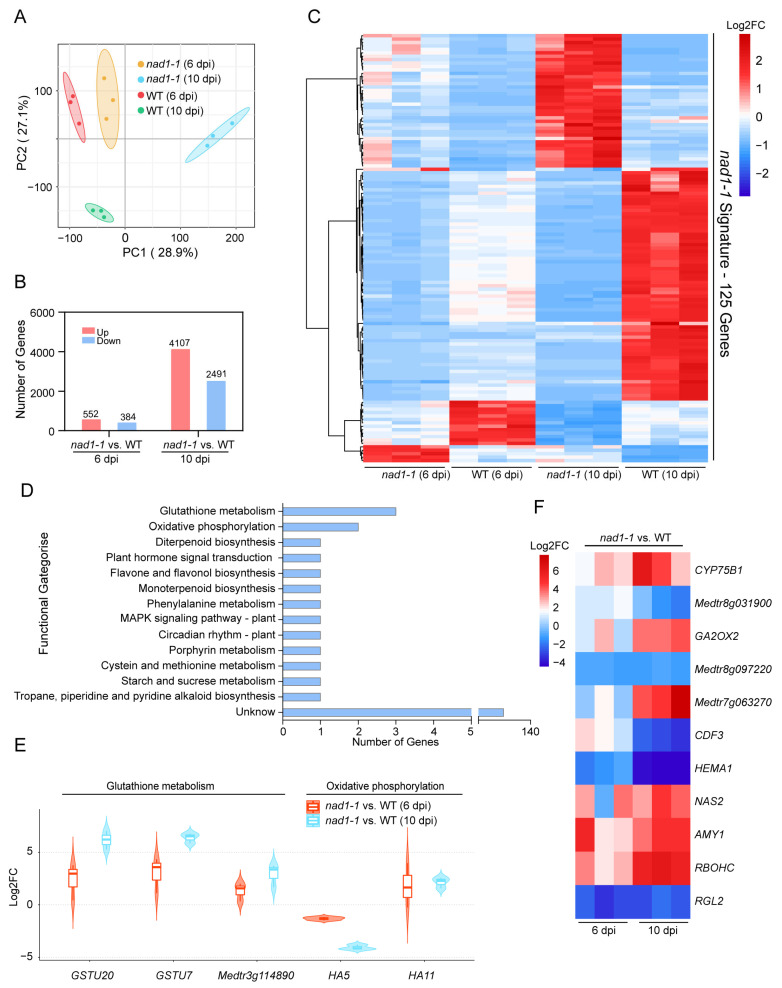
Identification of signature genes in WT and *nad1-1* mutant. (**A**) Principal-component analysis (PCA) of the WT and *nad1-1* transcriptome from two different times: *nad1-1* 6 dpi, wild type (WT) 6 dpi, *nad1-1* 10 dpi, wild type (WT) 10 dpi. (**B**) Number of differentially expressed genes (DEGs) in *nad1-1* vs. WT at 6 dpi and 10 dpi. (**C**) Heatmap showing relative expression levels for the ‘‘*nad1-1* signature’’, a set of 125 genes significantly regulated between *nad1-1* vs. WT at 6 dpi, and 10 dpi in *M. truncatula*. (**D**) KEGG pathway enrichment analyses of the “*nad1-1* signature” genes. (**E**) Violin plot showing expression levels of genes involved in the glutathione metabolism and oxidative phosphorylation pathways. (**F**) Heatmap showing relative expression levels for the genes in the other pathways (**D**). Gradient scale represents expression levels, with red showing the highest expression to blue with the lowest expression. “*nad1-1* vs. WT” represents comparison of transcription of gene families in *nad1-1* mutant nodules (*nad1-1*) vs. WT nodules (WT).

To explain the detailed mechanisms of how hosts interact with symbionts in the *M. truncatula nad1* nodules, we performed a dual RNA-seq to examine gene expression patterns in the *M. truncatula nad1* nodules at 6 dpi and 10 dpi. Dual RNA-seq data revealed the differences in transcriptional signatures between different development stages (6 dpi and 10 dpi) of nodules (Figure 2). Principal component analysis (PCA) of plant gene expression profiles at 6 dpi and 10 dpi was consistent with the phenotype between *nad1-1* mutant and wild-type nodules. Plant gene expression profiles of the *nad1-1* mutant nodules at 6 dpi were slightly different from the wild-type nodules (Figure 2A). The *nad1-1* mutant nodule profiles at 10 dpi were most different from the wild-type nodules (Figure 2A). According to the thresholds of fold change > 2, adjusted *p*-value < 0.05, we identified DEGs in the comparison of *nad1-1 mutant* nodules vs. wild-type (WT) nodules. A total of 936 plant DEGs were obtained (Table S1), of which 552 DEGs were up-regulated and 384 DEGs were down-regulated in *nad1* nodules at 6 dpi (Figure 2B). It should be noted that at 10 dpi, there was a dramatically increased number of DEGs (6598) (Table S1), of which 4107 and 2491 DEGs were up-regulated and down-regulated, respectively (Figure 2B). Among all DEGs identified, 125 DEGs were shared by those identified in nodules at both 6 dpi and 10 dpi (Figure 2C). We then defined these shared 125 DEGs as the “*nad1-1* signature” (Figure 2C).

The function of the “*nad1-1* signature” DEGs identified was investigated using KEGG pathway analysis (Figure 2D). The results showed that these “*nad1-1* signature” DEGs were mainly enriched in glutathione metabolism, oxidative phosphorylation, and flavonoid biosynthetic pathways (Figure 2D). Notably, 3 “*nad1-1* signature” genes including *GSTU20* and *GSTU7* in the glutathione metabolism were significantly up-regulated in *nad1* nodules at 6 dpi and 10 dpi (Figure 2E), suggesting that GSTU20 and GSTU7 might play important roles in suppressing immunity to support rhizobium survival in nodules, which is consistent with the biochemical function of these two genes in antioxidant reactions to eliminate the accumulation of ROS and lipid peroxides in infected tissues [42,43].

Plasma membrane H^+^-ATPase (P-type H^+^-ATPase) is a protein with a molecular weight of about 100 kDa, which is considered the exclusive protein of plant and fungal plasma membranes. This protein is localized on the biofilm to form an electrochemical gradient. P-type H^+^-ATPase can supply energy for metabolite absorption and responses to the environment [44]. In our study, the *HA5* (one type of P-type H^+^-ATPase) was up-regulated (Figure 2E), possibly providing energy for mutants to respond to rhizobial stress.

Flavonoids are secondary metabolites widely distributed in plants. As a flavonoid 3′-hydroxylase, *CYP75B1* belongs to the cytochrome P450 monooxygenase family, and it participates in the flavonoid metabolic pathway [45]. More importantly, the expression of *CYP75B1* can induce flavonoid biosynthesis to regulate the ROS homeostasis [46]. In this study, among the genes regulated at 6 dpi and 10 dpi, *CYP75B1* was markedly up-regulated potentially to remove reactive oxygen species (ROS) so as to weaken the immune response caused by *NAD1* protein deficiency in *nad1-1* mutant plants (Figure 2F). Taken together, the above data suggest that these DEGs identified at 6 dpi and 10 dpi in plants may be related to plant metabolism and immunity.

### 2.3. Identification of DEGs from Symbionts in the M. truncatula nad1 Nodules

**Figure 3:**
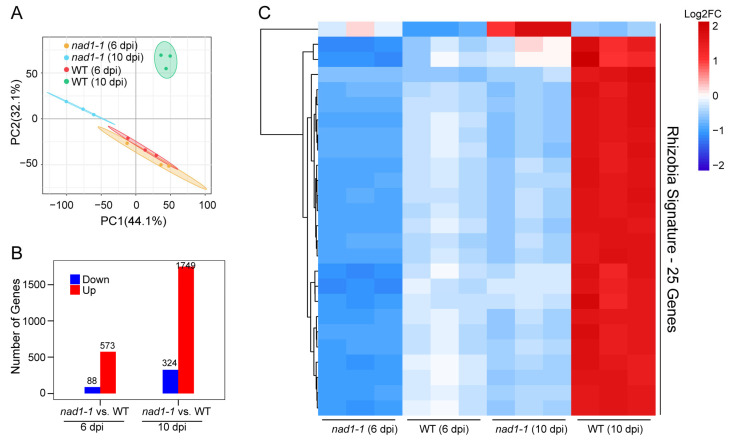

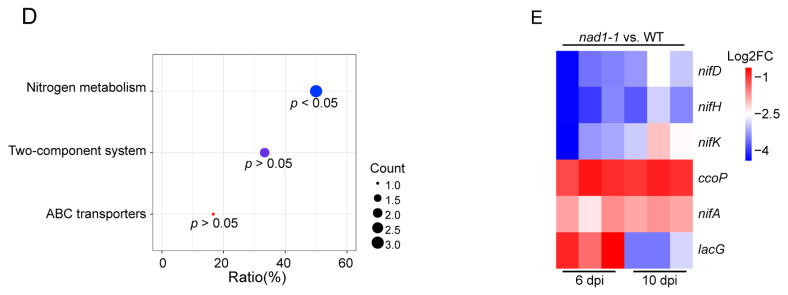
Analysis of different rhizobia transcriptional patterns in *nad1-1* vs. WT. (**A**) Principal-component analysis (PCA) of the rhizobia transcriptome from two different times: *nad1-1* 6 dpi, wild type (WT) 6 dpi, *nad1-1* 10 dpi, wild type (WT) 10 dpi. (**B**) Number of rhizobia differentially expressed genes (DEGs) in *nad1-1* vs. WT at 6 dpi and 10 dpi. (**C**) Heatmap showing the relative expression levels of rhizobial genes for the “Rhizobia signature”, a set of 25 genes significantly regulated in *nad1-1* vs. WT at 6 dpi and 10 dpi. (**D**) KEGG pathway enrichment analyses of the “Rhizobia signature” genes. The red and blue dots represent *p*-values. (**E**) Heatmap showing expression levels of genes involved in the nitrogen metabolism, two-component system, and ABC transporter pathways. Gradient scale represents expression levels, with red showing the highest expression to blue with the lowest expression. “*nad1-1* vs. WT” represents comparison of transcription of gene families in *nad1-1* mutant nodules (*nad1-1*) vs. WT nodules (WT).

We directly compared the gene expression profile of rhizobium between *nad1-1* mutant and wild-type (WT) nodules at 6 dpi and 10 dpi. PCA analysis of the rhizobium transcripts (Figure 3A) showed a very clear separation between *nad1-1* mutant and wild-type (WT) nodules at 10 dpi, but no obvious separation at 6 dpi. A total of 661 rhizobial genes at 6 dpi (573 up-regulated, 88 down-regulated), were found to be differentially expressed (fold change > 2, adjusted *p*-value < 0.05) in comparison of *nad1-1* mutant vs. WT (Figure 3B, Table S2), while 2073 genes were differently expressed at 10 dpi, of which 1749 genes were up-regulated and 324 genes were down-regulated (Figure 3B, Table S2).

In total, 25 rhizobial DEGs were shared at 6 dpi and 10 dpi, which was defined as the “rhizobium signature” (Figure 3C). In addition, we performed a KEGG pathway enrichment analysis of these rhizobium signature genes to reveal their functions. The results showed that three rhizobium signature genes were enriched in nitrogen metabolism, two in a two-component system, and one in the ABC transporter pathways during two stages (6 dpi and 10 dpi) of nodule development (Figure 3D). The expression levels of four nitrogen fixation-related genes (*nifA*, *nifD*, *nifH*, and *nifK*) were significantly down-regulated at both 6 and 10 dpi (Figure 3E), which was consistent with defective nitrogen fixation phenotypes in the *nad1-1* mutant nodules (Figure 1B). These data suggest that the DEGs identified at 6 dpi and 10 dpi in rhizobium might be related to nitrogen fixation in nodules.

### 2.4. Expression of Genes Associated with the NF Signaling Process

**Figure 4:**
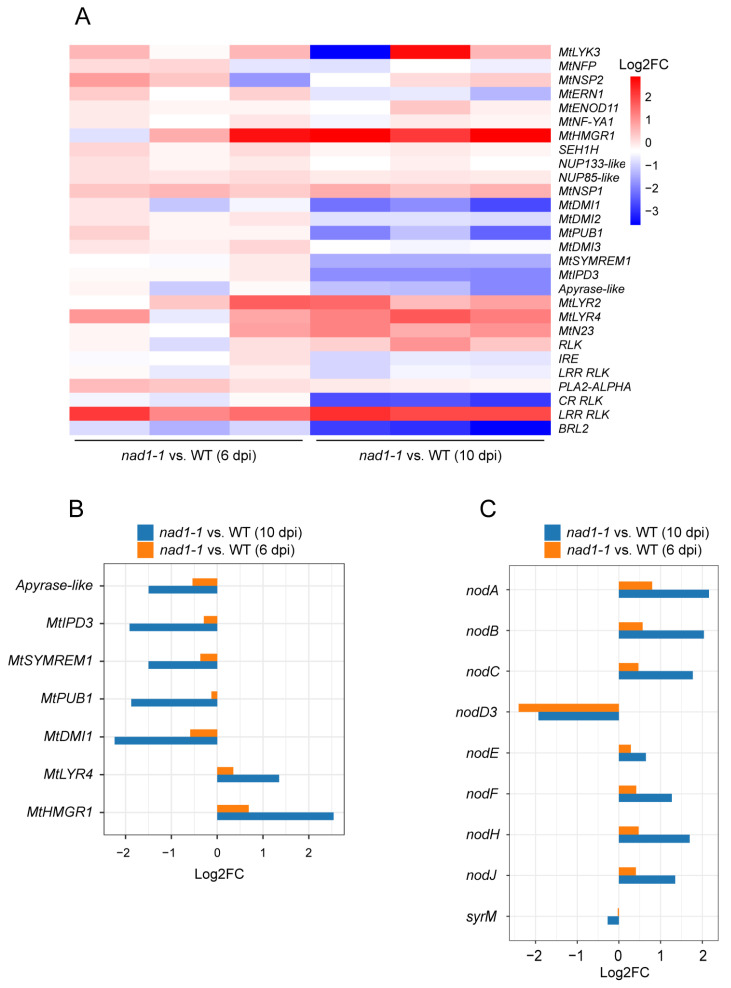
Candidate genes involved in the control of nod factor (NF) signaling. (**A**) Heatmap showing the transcriptional expression levels of all plant genes related to nodule NF signaling. Gradient scale represents expression levels, with red showing the highest expression to blue with the lowest expression. (**B**) Bar plot showing the expression levels of NF signaling-related genes regulated between *nad1-1* and WT at 6 dpi and 10 dpi in *M. truncatula*. (**C**) Boxplot showing the expression levels of NF-genes regulated in *nad1-1* vs. WT at 6 dpi and 10 dpi in *S. meliloti*. “*nad1-1* vs. WT” represents comparison of transcription of gene families in *nad1-1* mutant nodules (*nad1-1*) vs. WT nodules (WT).

NF production and signal transduction take place not only in the nodule infection zone but also in the nitrogen-fixing zone which might be associated in particular with infection threads [47]. In *M. truncatula*, *NAD1* is mainly expressed in interzone II–III of nodules [39]. To investigate whether *NAD1* is involved in NF signaling, we investigate the expressions of NF signaling-related genes (Figure 4A, Table S3). The expression levels of early-stage NF signaling-related *Medicago* genes including *MtLYK3, MtNFP, MtDMI2, MtERN,* and *MtNSP2* exhibited no difference between *nad1-1* mutant and wild-type nodules at 6 dpi or 10 dpi (Figure 4A), which suggested that *NAD1* may not be involved in early-stage NF signaling and rhizobial infection. Root nodule development-related genes including *MtIPD3*, *MtDMI1*, *Apyrase-like*, *MtSYMREM*, and *MtPUB1* were down-regulated in nodules at 10 dpi (Figure 4B). LysM receptor *MtLYR4* and 3-Hydroxy-3-Methylglutaryl CoA Reductase 1 (*HMGR1*) showed little difference between *nad1-1* mutant and wild-type nodules at 6 dpi, but their expression levels were significantly up-regulated in nodules at 10 dpi (Figure 4B), which is consistent with the phenotype observed in the nitrogen-fixing zone where a majority of cells have ruptured, as compared to the wild-type nodules at the same time point. Taken together, the above data implied that *NAD1* might not be involved in the NF signaling pathway, which is consistent with its nodule-specific expression pattern [39].

In terms of rhizobia, NF biosynthesis genes including *nodABC*, *nodF*, *nodH*, and *nodJ* exhibited little expression difference between *nad1-1* mutant and wild-type nodules at 6 dpi. However, their expression was significantly higher in *nad1-1* mutants than in wild-type nodules at 10 dpi (Figure 4C, Table S4). The gene expression level of transcriptional regulator *nodD3* belonging to the LysR family [48] was significantly down-regulated in nodules at both 6 dpi and 10 dpi (Figure 4C). Instead, another transcriptional regulator, *SyrM*, an activator for *nodD3* expression [48], displayed no difference between *nad1-1* mutant and wild-type nodules at either 6 dpi or 10 dpi (Figure 4C). These results indicated that *NAD1* might play a role in the late stages of nodule development by affecting the expression level of root nodule-related genes.

### 2.5. Expression of Genes Involved in Nodule Meristem and Differentiation

**Figure 5:**
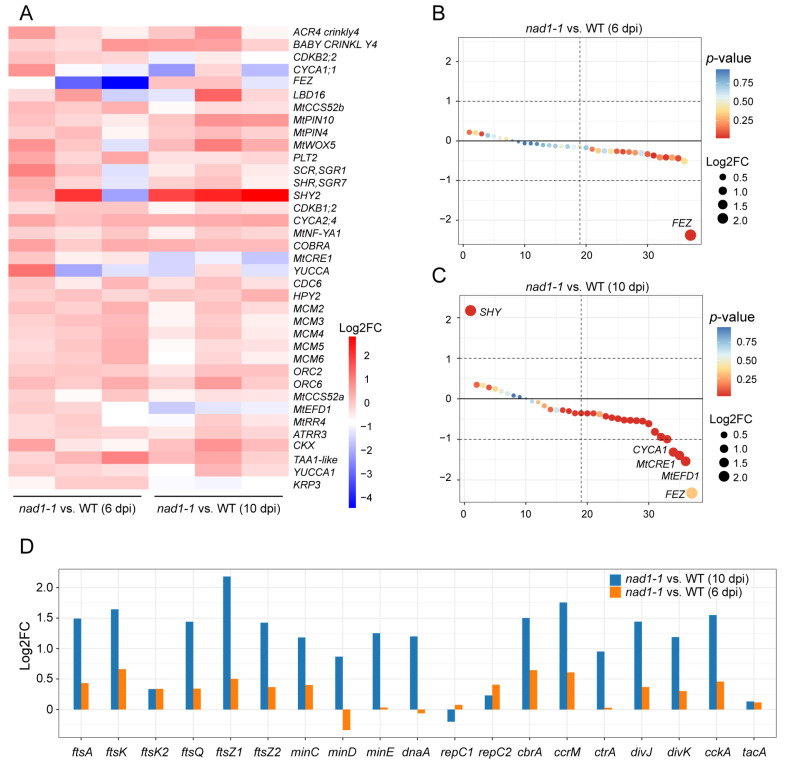
Candidate genes involved in the control of the nodule meristem and differentiation. (**A**) Heatmap showing the transcriptional expression levels of all plant genes related to nodule meristem and differentiation. Gradient scale represents expression levels, with red showing the highest expression to blue with the lowest expression. (**B**,**C**) Gene ranking dot plots showing the DEGs related to nodule meristem and differentiation between *nad1-1* and WT at 6 dpi (**B**) and 10 dpi (**C**) in *M. truncatula*. (**D**) Bar plot showing the genes related to nodule meristem and differentiation at 6 dpi and 10 dpi in *S. meliloti*. “*nad1-1* vs. WT” represents comparison of transcription of gene families in *nad1-1* mutant nodules (*nad1-1*) vs. WT nodules (WT).

In order to determine whether *NAD1* played a role in nodule formation and development, we examined the expression levels of genes related to nodule meristem and differentiation (Figure 5A,D, Tables S5 and S6) [47]. The results showed no significant difference in the expression of genes related to the nodule meristem and differentiation between *nad1-1* mutant and wild-type nodules at 6 dpi (Figure 5A,B), which implies that *NAD1* had no effect on the formation of nodule organogenesis at the early stage of rhizobial inoculation. However, the *SHY2* gene involved in cell differentiation was significantly up-regulated at 10 dpi (Figure 5C). Moreover, two cell differentiation-promoting genes *MtCRE1* [49], cytokinin (CK) receptor gene, and *MtEFD* [50] encoding an APETALA2/ETHYLENE RESPONSIVE FACTOR (ERF) transcription factor in nodules were down-regulated in nodules at 10 dpi (Figure 5C), which is consistent with the arrested development of *nad1-1* nodules at the late stage of nodule development.

For rhizobia, the process of terminal differentiation of bacteroids is associated with changes in rhizobial genes related to cell division during nodule differentiation [12]. Multiple genes from bacteria have been identified as regulating cell division or the cell cycle. Bacterial *ftsQAZ*, *ftsK*, and *minCDE* are known to be involved in cell division [51,52,53,54]; *dnaA*, *repC1*, and *repC2* are involved in the initiation of DNA replication [55,56,57], while *ctrA*, *cbrA*, *tacA*, *ccrM*, and *divJ* control the cell cycle [58,59,60,61]. The expression of the aforementioned genes was detected at maximal levels in FIId and FIIp but at significantly decreased levels in the IZ or ZIII [47]. In our study, most bacterial genes involved in cell division (*ftsQAZ*, *ftsK*, and *minCDE*), initiation of DNA replication (*dnaA*, *repC1*, and *repC2*), and cell cycle control (*ctrA*, *cbrA*, *tacA*, *ccrM*, and *divJ*) exhibited no significant difference in expression levels from those in wild-type nodules at 6 dpi (Figure 5D, Table S6). However, the expressions of the genes involved in cell division (*ftsA*, *ftsK*, *ftsQ*, *ftsZ1*, *ftsZ2*, *minC*, and *minE*), initiation of DNA replication (*dnaA*) and cell cycle control (*cbrA*, *tacA*, *ccrM*, *divJ*, and *cckA*) were significantly higher in *nad1-1* mutants than in wild-type nodules at 10 dpi (Figure 5D). The above data indicated that *NAD1* is required for the transition of bacteria from active division to a state of cell differentiation, undergoing multiple rounds of endoreduplication without division. Overall, these data suggest *NAD1* may affect the presence of rhizobium in the nodules, which is consistent with the *NAD1* function in the requirement of rhizobial colonization in symbiotic cells [39].

### 2.6. Effects of NAD1 on Nodule Symbiosis

**Figure 6:**
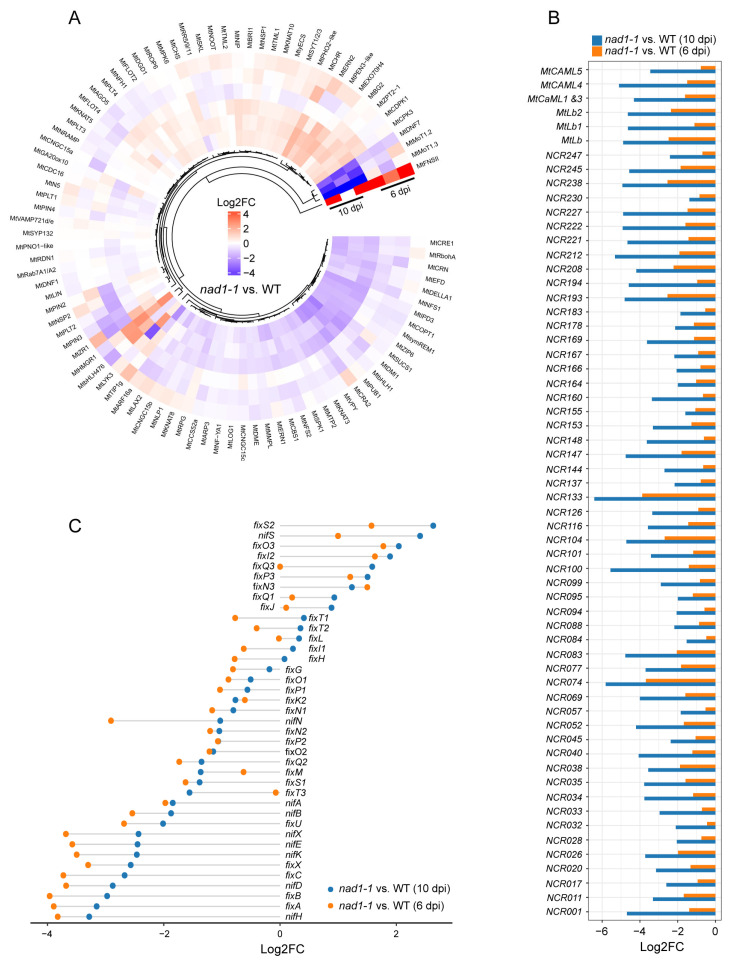
Identification of plant and rhizobial transcriptional responses in symbiotic nitrogen fixation processes. Gradient scale represents expression levels, with red showing the highest expression to blue with the lowest expression. (**A**) Heatmap showing relative expression levels for the genes in symbiotic nitrogen fixation processes in *M. truncatula*. (**B**) Barblot showing expression profiles of leghemoglobin, NCR, and CaM-like genes in *M. truncatula*. (**C**) Dot chart plot showing expression profiles of *nif* and *fix* genes in *S. meliloti*. “*nad1-1* vs. WT” represents comparison of transcription of gene families in *nad1-1* mutant nodules (*nad1-1*) vs. WT nodules (WT).

During the developmental stage of nodule differentiation, a distinctive feature is the accumulation of specialized heme-containing proteins, such as leghemoglobins, which are present in pink color in the nitrogen-fixation zone [62]. Leghemoglobins play crucial roles in creating microoxic conditions and are essential for nitrogenase activity and the maximal expression of rhizobial *nif* genes [62,63]. NCR peptides have been mainly found in the inverted repeat-lacking clade (IRLC) legumes, controlling bacterial growth and terminal bacteroid development [64,65]. Our data showed that the *nad1-1* mutant nodules exhibited reduced nitrogen fixation ability compared with wild-type nodules (Figure 1C). In order to explore the role of *NAD1* in the interaction between plants and rhizobia, we examined the expressions of the genes related to nodule symbiosis (Figure 6A,B, Table S7) [20]. Clusters of symbiotic genes such as leghemoglobin, NCR genes, and CaM-like genes are highly expressed in the nitrogen-fixation zone, interzone II-III, or within infected cells of the nitrogen-fixation zone in wild-type nodules at the late-stage of nodule development [62,66,67]. Remarkably, our data showed the expression levels of leghemoglobin, NCR, and CaM-like genes were significantly down-regulated in *nad1-1* nodules at both 6 dpi and 10 dpi compared to wild-type nodules (Figure 6B). This is related to the defect of nitrogen fixation activity in *nad1-1* mutant nodules. 

As for rhizobia, a striking feature in nitrogen-fixing is the expression of *nif* genes that encode nitrogenase complex [64]. In *Sinorhizobium* spp., *nif* gene expression is regulated by the FixL/J-oxygen sensing system and NifA transcriptional activator under microoxic conditions [64]. Among all the *nif* genes identified, NifA is a positive regulator of other *nif* genes and *fix* genes, while *fixL* and *fixJ* also regulate *nifA* gene expression. *nifHDK* is the structural gene of nitrogenase, which is highly conserved in all nitrogen-fixing microorganisms and induced by NifA. *FixL* and *fixJ* are a pair of regulator genes, and they can induce *nifA* and *nifK* gene expression and regulate symbiotic nitrogen fixation in root nodules through these two genes [68].

We observed significant changes in *nif* and *fix* gene expressions during nodule development (Figure 6C, Table S8). The expressions of six colocalized chromosomal genes (*fixs2*, *fixO3*, *fixI2*, *fixQ3*, *fixP3*, and *fixN3*) were significantly higher in *nad1-1* mutant nodules than in wild-type nodules at 6 dpi and 10 dpi (Figure 6C), which was different from the down-regulated expression pattern in the *dnf* mutant [64]. The iron-sulfur cluster assembly gene *nifS* exhibited a higher expression in *nad1-1* mutant nodules than in R108 nodules (Figure 6C), which suggested that *NAD1* might be involved in the formation of the nitrogen-fixation nodule. Moreover, half of the *nif* and *fix* genes (20 out of 40) exhibited lower expression in *nad1-1* mutant nodules than in R108 nodules (Figure 6C). The expression levels of these nitrogen-fixing genes were consistent with the phenotype of the *nad1-1* mutant that cannot fix nitrogen. It was noted that the expression of *fixL2* and *fixJ* was up-regulated at 10 dpi in the *nad1-1* mutant nodules (Figure 6C). Because FixJ/FixL is a major regulator of microoxic [69], bacteria might be exposed to microoxic conditions in the *nad1-1* mutant nodules. The expression patterns of *nif* and *fix* genes in this study also support the conclusion.

### 2.7. Interactome Analysis of Regulation of Nodulation by Plant Immunity

As an extraneous microorganism, the invasion of rhizobia tends to trigger certain plant immunity. During the early-stage symbiotic interactions, a weak immune response in plants is activated but gradually suppressed to an optimized level in subsequent symbiotic interactions [32]. Although rhizobia are required symbionts in nodules, they must actively suppress or escape from the plant’s innate immune system so as not to be identified as foes by hosts. As hosts, plants have evolved multiple strategies to regulate their own defense systems to allow rhizobial infection, colonization, and differentiation and nodule organogenesis [41]. Thus, a balanced immune response between legumes and rhizobia is required for the development of nitrogen-fixing nodules in plants.

**Figure 7:**
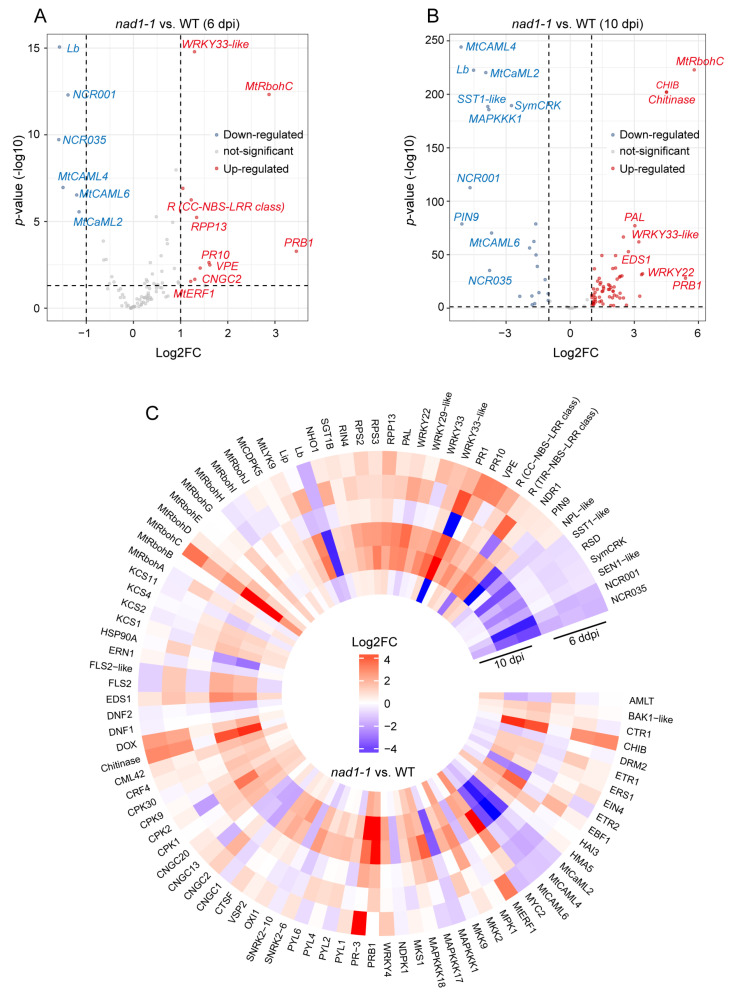
Transcriptomic analysis of *M. truncatula* gene expression during defense. (**A**,**B**), Volcano plots showing differential expression of defense genes in *nad1-1* vs. WT at 6 dpi (**A**) and 10 dpi (**B**). The top 10 genes ordered by log2 fold change are highlighted, and genes with an adjusted *p*-value of <0.05 are considered statistically significant. (**C**) Heatmap showing relative expression levels of defense-related genes in plant at 6 dpi and 10 dpi in *nad1-1* vs. WT. Gradient scale represents expression levels, with red showing the highest expression to blue with the lowest expression. “*nad1-1* vs. WT” represents comparison of transcription of gene families in *nad1-1* mutant nodules (*nad1-1*) vs. WT nodules (WT).

In order to reveal the role of *NAD1* in the interaction between plants and rhizobia, we investigated the expressions of the genes related to plant immune signals (Figure 7C, Table S9) at 6 dpi and 10 dpi. We identified 98 genes as plant immunity-related ones in *nad1-1* nodules, of which 10 genes were up-regulated and 18 genes were down-regulated in *nad1-1* mutants at 6 dpi (Figure 7A). At 6 dpi, the plant defense response was slightly induced in the *nad1-1* mutant. At 10 dpi, 64 genes and 25 genes were up-regulated and down-regulated in *nad1-1* mutant nodules, respectively, and the plant defense response was significantly induced (Figure 7B). Well-known marker genes related to defense such as *PR10* and *NDR1* were up-regulated at 6 dpi or 10 dpi (Figure 7A–C) and the genes involved in suppression of plant defense in nodules such as *DNF2*, *SymCRK*, and *RSD* were down-regulated in *nad1-1* mutants at 10 dpi, but their expressions exhibited little change at 6 dpi (Figure 7C). Furthermore, the accumulation of ROS also supported the occurrence of a defense response [70]. In plant systems, Rboh (NADPH oxidase) is one of the main systems that produce ROS. The expression of *MtRbohC* was significantly down-regulated both at 6 dpi and 10 dpi between *nad1-1* mutant and WT (Figure 7C). In addition, genes involved in PAMP perception such as LRR-receptor kinase (*FLS2*, *BAK1*) and LysM-receptor kinase (*MtLYK9*) showed significant upregulation in *nad1-1* mutant nodules at 10 dpi compared with WT nodules (Figure 7C). Similarly, the genes related to calcium channel (*CPK1*, *CPK2*, *CPK9*, *CPK30*, *CNGC1*, *CNGC2*, *CNGC13*, and *CNGC20*), MAPK pathway (*MKK2*, *MKK9*, *MAPKKK1*, *MAPKKK17*, and *MAPKKK18*) and WRKY transcription factors (*WRKY4*, *WRKY22*, *WRKY29-like*, and *WRKY33*) displayed little alteration at 6 dpi, but they were remarkably up-regulated at 10 dpi in *nad1-1* mutant, compared with those in wild-type nodules (Figure 7C).

A slight defense reaction including the defensin-like antimicrobial compounds was observed in the *nad1-1* mutant, which was in line with the expression change pattern of the defense-related genes (*PR10*, *NDR1*, R protein, *EDS1*, *MtRbohC*) at 6 dpi (Figure 7A). In this early-stage nodule development, the expressions of most genes related to symbiotic nitrogen fixation in plants exhibited little difference between the *nad1-1* mutant and wild type (Figure 6A). To escape from host defenses, the rhizobium-infected cells can passively protect themselves and actively modulate host functions [33]. Although there was little significant difference in the stress-related rhizobial gene expression between *nad1-1* mutant and wild type at 6 dpi, the *nif* and *fix* genes related to nitrogen fixation exhibited a significant difference at this stage (Figure 5C). One possible explanation is that a slight defense activation in the host may result in a significant reduction in bacterial *nif* and *fix* gene expression in nodules.

The *nad1-1* mutants made a strong defense response at 10 dpi, which was in agreement with the observation that most of the genes related to plant immunity and nitrogen fixation were significantly different between the *nad1-1* mutant and wild type. It has been reported that plants generate reactive oxygen species (ROS) as signaling molecules to participate in the legume–rhizobium symbiotic interaction [71]. Plant NADPH oxidase (NOX), also known as respiratory burst oxidase homolog (RBOH), is a key producer of reactive oxygen species (ROS) in plant immunity. In our study, RBOHC in *nad1-1* mutants showed significant up-regulation, compared to that in the wild-type group at both 6 dpi and 10 dpi, suggesting the involvement of MtRbohC-mediated ROS in defense responses in *nad1-1* mutant. Furthermore, knocking out the MtRbohBCD gene in the *nad1-1* mutants can weaken the defense responses of nodules [40], further confirming that MtRbohBCD-mediated ROS is involved in the defense response in nodules.

**Figure 8:**
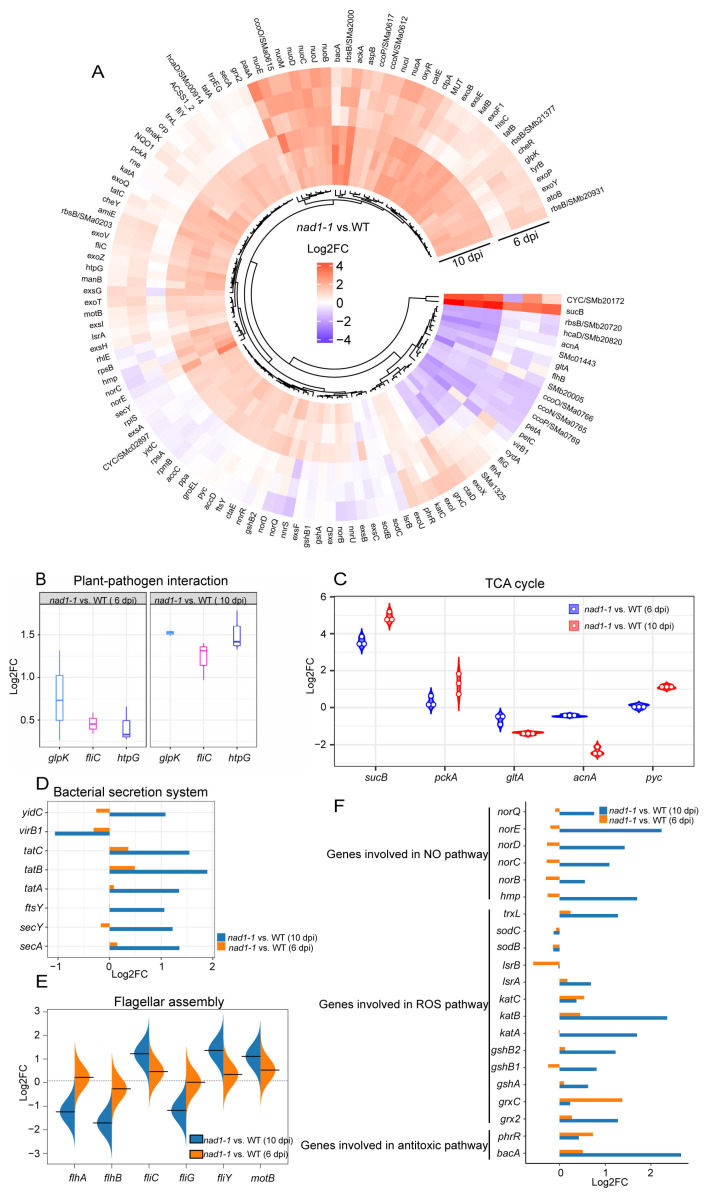
Several pathways are regulated during defense in *S. meliloti*. (**A**) Expression profiles of defense-related genes at 6 dpi and 10 dpi in *nad1-1* vs. WT. Gradient scale represents expression levels, with red showing the highest expression to blue with the lowest expression. (**B**) Boxplot of the main genes related to the plant-pathogen interaction pathway. (**C**) Violin plot showing expression levels of genes involved in the TCA cycle pathway. (**D**,**F**) Bar plots showing relative expression levels for genes in the bacterial secretion system (**D**), NO, ROS, and NCR pathways (**F**). (**E**) Bean plot showing expression levels of genes involved in the flagellar assembly. “*nad1-1* vs. WT” represents comparison of transcription of gene families in *nad1-1* mutant nodules (*nad1-1*) vs. WT nodules (WT).

Bacterial polysaccharides appear to be altered at later developmental stages and may play a role in suppressing plant defense responses and maintaining the symbiosis [72,73]. To reveal the response of rhizobia to the strong defense of *nad1-1* mutant plant, we investigated the expression of the genes involved in exopolysaccharide (EPS) synthesis and host defense suppression (*exoB*, *exoF*, *exoP*, *exoQ*, *exoV*, *exoY*, *exoZ*, *exsA*, *exsE*, and *exsG*) in the interactions between *M. truncatula* and *S. meliloti* [74]. The results showed that the expression of these genes showed no significant difference between *nad1-1* mutant and the wild-type nodules at 6 dpi, but their expressions were up-regulated at 10 dpi (Figure 8A, Table S10), which is consistent with the phenomenon of immune response being activated in *nad1-1* mutant nodules at 10 dpi. However, the precise mechanisms by which they act are unknown, future relevant experiments can be conducted to explore.

In the plant-pathogen interaction pathway, glycerol kinase-encoding *glpK* [75] and high-temperature protein G-encoding *htpG* [76] can influence nodule development and nitrogen fixation. In this pathway, *glpK* and *htpG* exhibited a higher expression level in *nad1-1* mutants than in the wild type at 10 dpi (Figure 8B). The tricarboxylic acid cycle (TCA cycle) plays a central role in maintaining bacterial metabolic status [77], and TCA cycle-related gene expression may change in *nad1-1* mutants. Consistent with this previous report, our results indicated that *sucB* encoding malate dehydrogenase in the TCA cycle was significantly up-regulated at both 6 dpi and 10 dpi (Figure 8C). Furthermore, TCA cycle-related genes (*gltA*, *acnA*, and *pyc*) exhibited higher expression levels in *nad1-1* mutant at 10 dpi (Figure 8C). Based on these findings, the high expression levels of TCA-cycle genes in *nad1-1* mutant nodules of defective nitrogen-fixation suggest that the rhizobia have access to plant-derived carbon sources, even though they do not efficiently fix nitrogen, which is consistent with previous research [64].

The bacterial secretion system is important for the virulence of many animal and plant pathogenic bacteria, and the rhizobial secretion system can secrete effectors to modulate their host specificity and symbiotic efficiency [33]. *SecA* and *SecY* are conserved and essential proteins for all bacteria, which are peripheral membrane ATPases that are involved in pre-protein translocation and integrated into the cellular membrane in bacteria [78]. Genes (*tatA*, *tatB*, and *tatC*) encoding translocase protein in a *SecA*- and *SecY*-independent manner are required for symbiotic nitrogen fixation and aspartate catabolism [79]. Our data showed that the genes related to protein transport mentioned above were significantly higher in *nad1-1* mutant than in wild type at 6 dpi and 10 dpi (Figure 8D), and that the expression of *virB1*, a component of the type IV secretion system, was down-regulated at both 6 dpi and 10 dpi (Figure 8D).

Since the flagellar assembly is crucial for the rhizobium motility and can influence nodulation [80], we examined the expression of flagella-related genes to explore the mobility of rhizobia. The results showed, the expressions of flagellar assembly genes (*flhA*, *flhB*, *fliC*, *fliG*, *fliY*, and *motB*,) showed little difference between *nad1-1* mutant and wild type at 6 dpi, but genes (*flhA*, *flhB*, *fliG*) and genes (*fliC*, *fliY*, *motB*) were remarkably down-regulated and up-regulated at 10 dpi (Figure 8E), respectively.

Bacteria are exposed to different environmental stresses including ROS and reactive nitrogen species during legume–rhizobial symbiosis [33]. The microsymbionts containing a large number of antioxidants and ROS-scavenging enzymes can protect the bacteroids against NO and ROS damage [33,64,65,81]. NO may participate in regulating the activity of two N_2_ fixation genes, *nifA* and *fixK*, by forming a complex with the membrane-bound protein FixL [82]. Increased NO level in nodule inhibits the expression of the rhizobial nitrogenase genes *nifD* and *nifH* [82,83]. Additionally, NO can also induce the expression of cytokinin receptor *CRE1* [84]. Elevated levels of NO within nodules are obtained through the utilization of *S. meliloti* mutant strains (*hmp*, *norB*, *nnrS1*) with impaired NO degradation [85]. Conversely, *S. meliloti* mutants overexpressing *hmp* have a reduced level of NO [86]. ROS is produced throughout root nodule development, acting as antimicrobial agents and signals for nodule organogenesis [71]. In the early phases of root nodule development, it is crucial to limit ROS levels, enabling the coexistence of rhizobia [71]. Higher ROS levels can have a negative impact on the survival of rhizobia [39,40].

In our study, genes enriched in NO pathway (*norBCD*, *norE*, *norQ*, and *hmp*) and ROS pathway (*trxL*, *katA*, *katB*, *gshA*, *gshB*, and *grx2*) displayed higher expression in *nad-1* mutant than wild type at 6 dpi and 10 dpi (Figure 8F). However, the balance of NO and ROS concentration between plants and rhizobia needs to be maintained to avoid its toxic effects, and its mechanisms during nodulation and nitrogen fixation processes remain unclear. Thus, functional tests of NO-related genes (*norB*, *norC, norD, norE, norQ*) and ROS-related genes (*trxL*, *katA*, *katB*, *gshA*, *gshB*, and *grx2*) in rhizobia may reveal whether they play a role in balancing symbiosis and immunity in nodules. To survive, bacteria exposed to toxic antibacterial compounds require the integrity of the BacA ABC-transporter [65]. The *bacA* mutant strain cannot differentiate and quickly dies after being released from the infection threads in nodules of *M. truncatula* [87]. Our results showed that the gene expression of *bacA* was significantly up-regulated in the *nad1-1* mutants at 10 dpi (Figure 8F), implying bacteria might display resistance to stress response mediated by antimicrobial compounds. 

Taken together, both plant and rhizobial genes exhibit transcriptional re-arrangements in the *nad1-1* mutant nodules, where the plant defense response is switched from an ‘inhibitory’ state to an ‘activated’ state.

## 3. Materials and Methods

### 3.1. Growth and Inoculation of Plant Cultivation, Inoculation, and Root-Nodule Harvest

Wild-type *Medicago truncatula* ecotype R108 and homozygous *nad1-1* mutants were employed for the phenotype and RNA-seq analysis. The *Tnt1* (for *transposable element of Nicotiana tabacum cell type 1*) insertion *nad1-1* mutant was isolated from the Noble Foundation (the ecotype R108 genetic background) *M. truncatula* mutant collection by a PCR-based reverse screening approach. In the *nad1-1* (NF18553) mutant, *Tnt1* insertion into the 5′-UTR region may lead to gene transcription inhibition. Seeds were subjected to scarification in H_2_SO_4_ for 2 min, followed by sterilization with 2.5% active chlorine for 5–8 min. Surface-sterilized seeds were synchronized at 4 °C in darkness for 2 days. Seeds were placed upside-down in N-free Fahraeus medium containing 1.2% agar, as specified in the Medicago Handbook (http://www.noble.org/MedicagoHandbook/, accessed on 20 December 2022). The growth experiments were conducted at 22 °C in darkness for a duration of 12–16 h to induce hypocotyl elongation. Germinated seedlings, numbering between nine and twelve, were transplanted into 10 × 10 cm growth pots. These pots contained a perlite: vermiculite mixture in a 1:2 ratio and were supplied with half-strength Fahraeus medium. The plants were cultivated under controlled conditions with a day: night regime of 16 h at 24 °C and 8 h at 18 °C, maintaining a relative humidity of 40–60%. Following four days of growth, each plant was inoculated with 50 mL of *Sinorhizobium meliloti* 2011 suspension per pot. The inoculum was prepared to an optical density at OD600 of 0.02. A liquid culture of *S. meliloti* 2011 was pelleted by centrifugation after overnight growth in Tryptone Yeast (TY) medium. The pellet was then resuspended in a half-strength Fahraeus medium containing 0.5 mM KNO_3_. Harvesting of R108 and *nad1-1* mutant nodules was performed for phenotype analysis and RNA-seq analysis 6 and 10 days after inoculation. For the purpose of RNA sequencing (RNA-seq), three independent biological replicates were prepared.

### 3.2. Acetylene Reduction Assay

The nitrogenase activity of nodulated roots, detached from intact plants, was measured using the acetylene reduction activity (ARA) method. These nodulated roots were obtained from plants 6 and 10 days after inoculation. This involved incubating three to five roots with 2 mL of acetylene (C_2_H_2_) in a closed 40 mL vial at 28 °C for 2 h. Acetylene gas was generated by reacting CaC_2_ (Sigma-Aldrich, St. Louis, MO, USA) with H_2_O and subsequently purified through filtration using a saturated CuSO_4_ solution. A volume of one hundred microliters of gas from each vial was utilized to measure the ethylene content employing a GC–4000A gas chromatograph (Dongxi, Beijing, China). For each sample, at least 40 plants divided into ten replicates were analyzed. Nitrogenase activity was calculated by normalization to nodule fresh weight and/or per plant. Statistical analysis was performed using GraphPad Prism version 6 with Student’s *t*-test; a probability value lower than 0.05 was deemed statistically significant.

### 3.3. Microscopy Analyses

For microscopy analysis, nodules were excised using a scalpel, allowing for slight trimming if necessary for aesthetics. The nodule surfaces were gently brushed clean using a small bristle brush. Then, harvested nodules were fixed using FAA (Servicebio, Wuhan, China) fixative solution and subjected to vacuum conditions for 30 min, followed by incubation at room temperature for 1–2 h. Subsequently, the nodules were rinsed twice with a phosphate buffer solution at pH 7.2, each time soaking for ten minutes.

The fixed nodules were dehydrated using a gradient of ethanol solutions (30%, 50%, 70%, and 100%). Each ethanol concentration was applied for 10–30 min, with three repetitions of the 100% ethanol step. The processed nodules were subjected to resin infiltration through the following procedure: sequential immersion in SolA with anhydrous ethanol ratios of 1:3, 1:1, and 3:1, each lasting 30 min to 1 h, followed by a 1 h immersion in SolA. SolA was composed of the following components: 100 mL of Technovit 7100 (KULZER, Hanau, Germany), 1 pack of Hardener I (KULZER, Hanau, Germany), and 2.5 mL of PEG400 (Sigma-Aldrich, St. Louis, MO, USA). Following this, the nodules were carefully positioned in molds, with a capacity of 3 to 5 nodules per mold. The embedding solution was introduced gradually along the contours of the mold to prevent nodule displacement. Subsequently, the molds were securely covered with sealing film. Ultimately, the molds were positioned within a fume hood to facilitate solidification. The embedding solution was prepared through the combination of 15 mL of SolA and 1 mL of Hardener II (KULZER, Hanau, Germany), with this preparation procedure being performed on ice. Resin-embedded nodules were sectioned longitudinally into 5 μm slices using a HistoCore AUTOCUT (Leica, Wetzlar, Germany). Nodule sections were stained with toluidine blue at room temperature for 30 min, followed by multiple rinses with distilled water using a small amount of water vapor each time. Sections were observed and photographed using a light microscope (Nikon ECLIPSE 80i, Tokyo, Japan). Nodules were observed and photographed using a fluorescence stereo microscope (Olympus SZX16, Tokyo, Japan).

### 3.4. RNA Isolation/RNA Purification, Amplification, and Sequencing

Samples for transcriptomic analysis were obtained from root nodules harvested 21 days after inoculation, placed in liquid nitrogen, and stored at −80 °C. For each of the three replicates, 9 to 17 plants (50–100 mg nodules) were used. Root nodules from *nad1-1* mutants and from wild-type cultivars R108 were placed in 750 μL LS Total RNA Extraction Reagent buffer (Yeasen, Shanghai, China) and frozen in liquid nitrogen. The nodules were ground with a high-throughput tissue homogenizer (SCIENTZ-48L, Ningbo, China) as the buffer thawed, then mixed and incubated for 5 min at RT to allow complete dissociation of the nucleoprotein complexes of the host cells. Specimens were then centrifuged at 4 °C, 12000× *g* for 10 min to remove more proteins, fats, and polysaccharides. A total of 80% of the LS Total RNA Extraction Reagent buffer (containing host RNA) was removed and placed in an RNase-free tube for later use. Then, 200 μL chloroform was added to the LS Total RNA Extraction Reagent buffer. The tubes were vigorously agitated for 15 s, and then placed at room temperature for 2–3 min. Specimens were centrifuged at 4 °C, 12,000 rpm for 10–15 min. The upper aqueous phase was carefully pipetted into a new centrifuge tube, and an equal volume of isopropanol was added. The tubes were inverted to mix and left at room temperature for 10 min. Specimens were then centrifuged at 4 °C, 12,000× *g* for 5 min. The supernatant was discarded. Samples were allowed to air-dry at room temperature for 5–10 min. Subsequently, 30–100 μL RNase-free water was added to dissolve the RNA. Isolated RNA samples were quality checked using an Agilent 2100 Bioanalyzer.

### 3.5. Library Preparation and Sequencing

The desired number of RNA samples was taken, and double-stranded and single-stranded DNA present in the RNA samples were degraded by DNase I (Yeasen, Shanghai, China). rRNA removal was performed using 10 ng–4 μg total RNA input. Streptavidin magnetic beads were used to remove rRNA based on the specific capture of ribosomal RNA sequences. The rRNA-depleted samples were purified by precipitating the RNA. The preprepared first-strand synthesis reaction mixture was added to the fragmented RNAs. The reaction program was set up for synthesizing first-strand cDNAs. The reaction system and program were configured and set up for second-strand cDNA synthesis, with dUTP used instead of deoxythymidine triphosphate (dTTP). After the reaction system and program were configured and set up, double-stranded cDNA fragments were subjected to end-repair, and then a single ‘A’ nucleotide was added to the 3′ ends of the blunt fragments. The reaction system and program for adaptor ligation were subsequently configured and set up to ligate adaptors with the cDNAs. Single-stranded circular DNA molecules were replicated via rolling cycle amplification, and a DNA nanoball (DNB) containing multiple copies of DNA was generated. DNBs of sufficient quality were then loaded onto patterned nanoarrays using the high-intensity DNA nanochip technique and were sequenced through combinatorial Probe-Anchor Synthesis (cPAS). The RNA-seq library was sequenced on a DNBseq platform with paired-end reads at Huada (Shenzhen, China). About 14 Gb of cleaned reads were obtained for each sample.

### 3.6. Dual RNA-Seq Data Analysis

The raw data with adapter sequences or low-quality sequences were filtered. A series of data processing steps were initially undertaken to remove contamination and obtain valid data. This step was accomplished using SOAPnuke (v1.5.6) software developed by BGI. The SOAPnuke software filtering parameters used were: “-n 0.01 -l 20 -q 0.4 -adaMR 0.25 -ada_trim -polyX 50 -minReadLen 150” [88]. Clean reads were mapped to the *M. truncatula* A17 genome (Mt20120830-LIPM) [47] and the *S. meliloti* genome GMI11495-Rm2011G.20130218.submit.genome.fna. Alignment and quantification were conducted using the STAR software (2.7.3a), leading to the generation of read count data. The gene expression level was measured by Fragments per Kilobase per Million Mapped Fragments (FPKM), the most commonly used method for estimating gene expression abundance. DEGSeq was used to analyze DEGs (fold change ≥ 2 and *p*-value ≤ 0.05). Principal component analysis (PCA), Gene Ontology (GO) term enrichment analysis, and Kyoto Encyclopedia of Genes and Genomes (KEGG) pathway enrichment analysis were performed using Omicshare, a real-time interactive online data analysis platform (http://www.omicshare.com, accessed on 15 August 2023). Heatmaps, bar plots, bean plots, box plots, violin plots, volcano plots, dot chart plots, and gene ranking dot plots were all carried out using HIPLOT PRO (https://hiplot.com.cn/cloud-tool/drawing-tool/list, accessed on 18 August 2023). The data that support the findings of this study have been deposited into the CNGB Sequence Archive (CNSA) of China National GeneBank DataBase (CNGBdb) with accession number CNP0004891.

## 4. Conclusions

This study provides a comprehensive transcriptome from both hosts and symbionts in the *M. truncatula nad1* mutant nodules, presenting a landscape of how hosts and symbionts simultaneously cooperate with each other to respond to plant immune responses. The dual RNA-seq transcriptome technology is a powerful tool for the simultaneous assay of both rhizobial and plant gene expression within the context of nodule development. In *nad1* nodules at 6 dpi, when plant immunity is slightly activated, a slight induction of plant immunity-related genes and an obvious decrease in rhizobial nitrogen fixation genes were identified. While a striking immune response is activated in the *nad1* mutant nodules at 10 dpi, it does not lead to complete necrosis in nodules. Plant immunity-related genes are activated, leading to the over-accumulation of antibacterial compounds such as ROS and NO, accompanied by the expression of numerous genes responding to ROS and NO stresses in rhizobia. The expression changes of genes from both hosts and symbionts gradually shut down rhizobial colonization and nodule development. These data shed light on the indispensable role of *NAD1* in tapering off the plant immune response to gradually progress bacterial endosymbiosis in nodules, deepening our understanding of symbiotic nitrogen fixation and opening new avenues for enhancing symbiotic plant-microbe interactions in agricultural contexts.

## Data Availability

The data disclosed in this study can be accessed through the article or Supplementary Materials provided here.

## Supplementary Materials

The supporting information can be downloaded at: https://www.mdpi.com/article/10.3390/ijms242216178/s1.
